# External Comparator Cohort studies - clarification of terminology

**DOI:** 10.3389/fdsfr.2023.1321894

**Published:** 2024-01-24

**Authors:** Gerd Rippin, Joan Largent, Wilhelmina Elisabeth Hoogendoorn, Héctor Sanz, Jaclyn Bosco, Christina Mack

**Affiliations:** ^1^ IQVIA, Biostatistics, Frankfurt, Germany; ^2^ IQVIA, Epidemiology, Boston, CA, United States; ^3^ IQVIA, Epidemiology, Amsterdam, Netherlands; ^4^ IQVIA, Biostatistics, Barcelona, Spain; ^5^ IQVIA, Epidemiology, Boston, MA, United States; ^6^ IQVIA, Epidemiology, Research Triangle Park, NC, United States

**Keywords:** External Comparator studies, External Comparator Cohort studies, External Comparator Arm studies, Externally Controlled Trials, Historical Control studies, Synthetic Control studies, terminology, nomenclature

## Abstract

Though there is only one term for the gold standard of Randomized Controlled Trials the terminology used for controlled research involving external data is diverse. Common terms include External Comparator/Control Arm study, Externally Controlled Trial, Synthetic Control study and Historical Control study. The term Externally Controlled Trial was recently selected by the U.S. Food and Drug Administration (FDA) and is in line with the use case of a pivotal trial. It entails pre-specification of the external dataset and its analysis in the trial protocol, which produces the highest amount of transparency, which is an important aspect for maximum credibility. If this pre-specification did not occur, we advocate the term External Comparator Cohort study (or short External Comparator study), which is derived by scrutinizing the paired terms study/trial, control/comparator and arm/cohort. Furthermore, we propose an overall framework of nomenclature, which is generally applicable for research projects involving external data. Only a precise and consistent use of terminology will most effectively safeguard from unintended implications, inaccurate perceptions, and misguided mindsets.

## 1 Introduction

The Randomized Controlled Trial (RCT) paradigm is paramount for drug approval studies. There is wide agreement that the RCT design should be chosen for such studies when feasible and ethical. The RCT design incorporates an internal control group that minimizes confounding from baseline factors, while the concept of utilizing external data as a comparison cohort to the trial population comes with methodological challenges, which have been well-described in the literature (Skovlund et al., 2018; Seeger et al., 2020; Burger et al., 2021; Rippin et al., 2022; Ghadessi et al., 2020). Though re-discussing details of these limitations is out of scope of this publication, it is helpful to acknowledge methodological and practical limitations from a general standpoint. This helps establishing valid use cases, which are related to situations where a large treatment effect is anticipated (U.S. Food and Drug Administration, 2018). A guideline of when to choose which kind of control group is available since 2001 (European Medicines Agency, International Council on Harmonization, 2001), while a reflection paper about submitting Single-Arm Trial (SAT) results as pivotal evidence (European Medicines Agency, 2023), and a dedicated (draft) guideline specific to the Externally Controlled Trial (ECT) approach (U.S. Food and Drug Administration, 2023) became available only recently.

This publication aims to clarify nuances in nomenclature when using external data, which seems to be timely given the recently issued draft U.S. Food and Drug Administration (FDA) guidance (U.S. Food and Drug Administration, 2023) mentioned above and the International Council of Harmonisation (ICH) draft reflection paper on the proposed international harmonization of real-world evidence (RWE) terminology (European Medicines Agency, International Conference of Harmonisation, 2023). We examine whether applied terminologies are exchangeable or meaningfully different and discuss whether some nomenclature should be considered preferable over others. Specifically, Section 2 starts with scrutinizing the three paired terms trial/study, control/comparator and arm/cohort to derive the most suitable terms for classes of research projects involving external data sources. The Section discusses further nomenclature as well, including the composite terms External Comparator/Control Arm studies, Externally Controlled Trials, Synthetic Control studies, and Historical Control studies. It is shown that only a precise use of terminology will most effectively safeguard from unintended implications, inaccurate perceptions and misguided mindsets. Section 3 concludes the presented terminology framework by providing a discussion.

## 2 Clarifications and nuances in nomenclature

For a summary of the discussed terms in this Section see Table 1.

**TABLE 1:** Terms and clarifications.

Term	clarification
Trial	Clinical trial, involving treatment interventions
Study	Observational study, no treatment interventions
Control	Control treatment/patients. Alignment to clinical trials possible, but not implemented in important guidelines
Comparator	Comparator treatment/patients. Alignment to observational studies possible, but not implemented in important guidelines
Treatment Arm	Clinical trial terminology denoting the treatment group
Treatment Cohort	Observational study terminology denoting the treatment group
Externally Controlled Trial (ECT)	Single-arm trial (SAT) utilizing external data for comparison purposes. In line with the use case of a pivotal trial, which requires pre-specification before starting the SAT for maximum credibility
External Comparator (Cohort) Study (EC Study)	As ECT, but no pre-specification. Involves a separate study protocol
Concurrent/Contemporaneous	Similar data temporality across cohorts
Historical/Non-concurrent/Non-contemporary	Differential data temporality across cohorts
Historical Control Studies	Term describing comparisons with non-concurrent external patient data
Synthetic Control Studies	The original term describes a specific statistical method relevant for aggregated data. Sometimes used for ECTs/EC studies (discouraged)
Benchmarking using aggregated data	Comparison based on simple descriptive statistics (no sophisticated bias control)
Studies using individual patient-level data	Allows for sophisticated bias control by using complex statistical approaches

### 2.1 Clinical trials versus observational studies

The ICH Good Clinical Practice (GCP) Network defines the terms clinical trial and observational study as follows: “There are two main types of clinical studies: clinical trials (also called interventional studies) and observational studies“ (International Conference of Harmonisation (ICH) GCP network, 2023a). The definition separates both terms and associates a study being observational while in “a clinical trial, participants receive specific interventions according to the research plan or protocol created by the investigators (International Conference of Harmonisation (ICH) GCP network, 2023a)”. This ICH GCP clinical trial definition is supported by the definition of the National Institutes of Health (NIH), which states that in a clinical trial “one or more human subjects are prospectively assigned to one or more interventions (which may include placebo or other control) to evaluate the effects of those interventions on health-related biomedical or behavioral outcomes” (National Institutes of Health, 2018). The ICH glossary states that the use of the terms trial or study are equivalent when the term clinical is added: “The terms clinical trial and clinical study are synonymous.” (International Conference of Harmonisation GCP network, 2023b). However, clarity is enhanced of course by consistently using the term clinical trial to separate the design unambiguously from observational studies.

Research projects using external data are often set up as a hybrid clinical trial/observational study. For example, a Phase II SAT (which is a clinical trial) can be combined with external observational real world (RW) data. While it is clear how to label the two components of the design, it is a natural question whether this composite design should be labeled as a trial or a study. This question is addressed in Section 2.4.

### 2.2 Control versus comparator

The terms control and comparator are associated with a control/comparator treatment (which also can be placebo or standard of care) and control/comparator patients receiving these treatments (European Medicines Agency, International Council on Harmonization, 2001; Mack et al., 2020). Fully controlled research, however, requires additional dimensions. One is to control relevant features by detailed written protocol specifications, such as outcome measurement methods and timings or standardized baseline diagnostic methods, but this is not always possible to implement/mandate when using external data. Further, eligibility criteria are highly likely to not be fully identical across the two cohorts due to limited data availability and granularity in the external data source. Additionally, data generation is recommended to be controlled with the highest monitoring and data management standards. Also, measured baseline characteristics like data temporality, regions and site types may differ systematically, with potentially limited ability to control for these influences. While the statistical task of bias control is well achieved by randomization, non-randomized studies cannot rule out unmeasured confounding, as it is an untestable assumption. Similarly, post-baseline intercurrent events may differ more than in RCTs. Hence, the treatment effect estimation based on the treatment policy estimand strategy (ICH E9(R1) Expert Working Group, 2021), which considers the start of the treatment but not any intercurrent events (like treatment modifications, additions or discontinuations, and the effect of any subsequent therapies) can be more difficult to interpret compared to RCTs, which makes projects utilizing external data even less controlled. Thus, it is very probable that a design with control treatment/patients using external data is not able to fully control all aspects of the research. Thus, the question arises whether it is helpful to use the term control treatment or control patients when the experiment overall cannot be entirely controlled.

Traditionally, however, the term control is used for both internal and external controls, see, for example, the ICH E10 guideline about the choice for control groups in clinical trials (European Medicines Agency, International Council on Harmonization, 2001). This is also true for the new FDA draft guideline about Externally Controlled Trials (U.S. Food and Drug Administration, 2023). As described above, this is a perfectly valid approach, which interprets the term control/controlled merely as the existence of a control treatment. However, it is important to be aware that a design which uses control treatments/patients can still be uncontrolled in many aspects of the design. In this sense a nuanced nomenclature was previously suggested when to use the term control and when to use comparator (Rippin et al., 2022). When reserving the term control for internal control group settings only, control patients are defined to come from the control arm of the same RCT, while comparator patients are not. Comparator patients taken from a different RCT are still not labelled as control patients for the new study, but rather as comparator patients for the same reasons as outlined above.

When following the presented logic above, the difference between control and comparator patients is obviously very subtle, but it is our thinking that there is added value in using refined terminology to safeguard from incorrect expectations.

### 2.3 Arm versus cohort

In RCTs with two treatment groups, data fall quite naturally to the same body of evidence due to randomization, like arms belonging to the same physical body of a human being. The term arm signals a close and almost intimate connection, which is non-existent when using external data, as various external data sources may be candidates for a comparator cohort. It is suggested to label both groups as cohorts to avoid inaccurate perceptions. This proposal differs from FDA’s ECT draft guidance (U.S. Food and Drug Administration, 2023), where the term arm is used instead, taking over RCT terminology, which may lead to an incorrect mindset by thinking (maybe subconsciously) that there is more similarity to an RCT design than there actually is.

### 2.4 Externally Controlled Trials versus External Comparator Cohort studies

The term Externally Controlled Trials selected by the FDA in their recently issued draft guidance (U.S. Food and Drug Administration, 2023) is in line with the use case of a pivotal trial. Within the guidance the FDA states that for ECTs sponsors “should finalize the protocol before initiating the externally controlled trial […]”. All planning is performed upfront, which produces the highest amount of transparency, which is an important aspect for maximum credibility. Of course, staying consistent with FDA terminology is key, which leads to keep using the term Externally Controlled Trial if the requested condition of pre-specification is fulfilled. If it is not, we suggest the terms External Comparator Cohort (ECC) or External Comparator (EC) study, following the logic as laid out in Sections 2.1–2.3: The term comparator is employed as derived in Section 2.2 to protect from misperceptions when using the term control, since the study is not fully controlled overall. We prefer cohort instead of arm as explained in Section 2.3. Finally, the term study is applied (see Section 2.1), because the trial part was planned earlier under a separate protocol, while the new study comparing treatment effects outside the original trial protocol occurs in a setting where observational study research methods are applied. Even if the comparator cohort is taken from another trial, the intended comparison has not been pre-specified, such that the new research project is labelled as a study, which compares two different sources of trial data using observational research approaches like causal inference methods.

The ECT/ECC study differentiator of pre-specification usually translates to the existence of either one or two protocols. In case there is just one protocol including all the ECT design and analytical elements which need to be prespecified (as a minimum, selection of the external comparator cohort and the analytical approach, list of suitable data sources, eligibility criteria, appropriate exposure definitions and windows, endpoints, cogent analytic plans and approaches to minimize missing data and bias) (U.S. Food and Drug Administration, 2023), it is appropriate that the trial aspect is weighted highly, labeling the overall design as a trial. Of course, if there is complete pre-specification of the ECT, but two protocols are written (one for the trial and one for the study part), this would still constitute an ECT overall. Without pre-specification, on the other hand, there is a clear separation of the respective trial protocol and the later observational study protocol, and consequently there is no issue in labeling the different parts as trial and ECC study, unambiguously.

### 2.5 Historical versus non-concurrent

The ICH E10 guideline uses the terms concurrent and historical (European Medicines Agency, International Council on Harmonization, 2001). The term historical could be perceived to denote a negative connotation, which may have been intended when having the RCT paradigm as the gold standard in mind. However, outside of RCTs, historical (or non-concurrent) comparator patients may be highly valuable to increase the evidence for a new treatment, especially in rare diseases where recruitment can be challenging. In this case, including non-concurrent comparator patients may have a decisive positive effect on sample size reaching adequate statistical power.

The paired terms concurrent/non-concurrent (or contemporaneous/non-contemporaneous) seem to be a more neutral option to describe the study design, though the term historical is possible to use as well, as this is supported by important guidance documents (European Medicines Agency, International Council on Harmonization, 2001; U.S. Food and Drug Administration, 2023).

The practical use of terms regarding data temporality is likely to be vague and open to interpretations, which consequently may not offer added clarity. Researchers will have to decide whether a certain (small) difference in data temporality would make the comparison non-concurrent. However, terms based on interpretations/assessments are not necessarily helpful and may lead to additional confusion and disagreement. Moreover, the stability (or change) in the treatment landscape is typically the more decisive factor, rather than true data temporality concurrence. For example, if there is no change in the treatment landscape in the last 5 years, and the data reaches back for 5 years, the addition of the term historical or non-concurrent may be somewhat misleading. One could think of a more complex terminology like treatment-landscape concurrent, but it is debatable whether treatment-landscape concurrence qualifies for a binary category (yes/no), as there may be slight deviations in a practical study, for example, for a few of the participating countries.

### 2.6 Benchmarking using aggregated data versus causal inference using individual patient-level data

Another term which has been used for ECC studies is benchmarking (Mack et al., 2020), which is associated with a scenario where only aggregated data are available. A benchmark comparison may occur if there are restrictions delivering individual patient-level data (IPD), for example, due to legal or data privacy reasons. Such a situation is different from the ideal IPD setting, which allows for the best bias control, including the application of causal inference methods. Use of RW benchmarks for comparison purposes may nevertheless be a valuable way to attain contextual information about outcomes of patients external to the trial, especially if there are no existing results in the literature which could be referred to. Although patients are not combined in the same database, they still serve as an aggregate reference to the outcomes of the patients in the trial.

### 2.7 Other terminology when using external data

In addition to FDA’s ECT term which is in line with a pivotal trial and the newly advocated ECC study term in the case of no pre-specification (therefore operating under a separate study protocol), the literature has been using the terms External Comparator/Control Arm studies (Jaksa et al., 2022; Rippin et al., 2022), Synthetic Control studies (Thorlund et al., 2020), and Historical Control studies (Ghadessi et al., 2020; European Medicines Agency, International Council on Harmonization, 2001; U.S. Food and Drug Administration, 2023). While the first expression can be improved by consistently using the terms Comparator and Cohort, usage of the latter two expressions needs a more detailed discussion.

The term Synthetic Control study should not be applied for ECTs/ECC studies from our perspective, because it is already used for another kind of design, , which applies re-weighting units from aggregated data (Abadie and Gardeazabal, 2003; Boutell et al., 2018; Abadie, 2021). This method is utilized in economics and social sciences (Abadie, 2021) and in population-based health interventions occurring at an aggregate level (Boutell et al., 2018), originally proposed by Abadie and Gardeazabal, 2003. Also, when taking this term to describe ECTs/ECC studies, some readers may think that the comparator patients may somehow not be real patients (with synthetic potentially also being perceived to be negative) (Rippin et al., 2022). Though it was argued that the term Synthetic Control study denotes an “emergent set of methodologies” which “have been utilized to provide greater insight into external control data” (Thorlund et al., 2020), we think that it is more consistent to reserve the term Synthetic Control study to describe the original analysis approaches as described in Abadie, 2021, Boutell et al., 2018; Abadie and Gardeazabal, 2003. Also, the term does not seem to offer added value when comparing to the now “official” FDA ECT term and the ECC study term as derived in this paper.

The term Historical Control study is explained in the ICH E10 guideline as the comparator cohort consisting of “patients treated at an earlier time” (European Medicines Agency, International Council on Harmonization, 2001). It is a valid terminology due to the ICH E10 reference, but the nuances in connotation as described in Section 2.5 are leaning rather to abstain from using/adding a temporal attribute to the ECT/ECC study design. Furthermore, the composite term Historical Control study (like Synthetic Control study) includes the term control, which was considered to be less preferable than comparator (Section 2.2).

## 3 Discussion

There are benefits in clarifying terminology for research projects involving external data, especially the nuances of the 3 paired terms control/comparator, arm/cohort and trial/study. Those who are most familiar with conducting RCTs may use the terms control, arm and trial out of habit which may be misperceived to imply an inadequate proximity to the RCT setting when using external data. Re-using RCT nomenclature to the case of not fully controlled settings may affect (potentially subconsciously) unrealistic expectations of the design approach.

The term Externally Controlled Trial as selected by the FDA is associated with the special use case of pre-specifying the external comparison before the clinical trial starts, which is in line with the idea of a pivotal trial. On the other hand, the term External Comparator Cohort study was derived to be an adequate description of the more common use case of no pre-specification, which operates under separated clinical trial and observational study protocols.

While the ECT design is in line with a use case of a pivotal trial, ECC studies still offer added value in providing context to study findings, because they do not have to rely on expert opinions or literature approximations only (if available at all). Information from the literature may rely on eligibility criteria which are (potentially markedly) different and no individual patient-level comparator data is available for enabling best bias control methods. Of note, if an ECC study use case is being discouraged because of no pre-specification then all stakeholders could end up with less generated evidence by simply having to rely on the SAT only, while going the extra mile of performing an ECC study could have provided valuable and potentially crucial study-specific contextualization. Thus, the perception should be avoided that only ECTs are valid use cases for the general study design and a distinction of the terms ECT and ECC study should prove helpful to be conscious of the validity of the two different use cases.

The FDA importantly noted that ECTs/ECC studies may be considered “when the effect size is expected to be large” (U.S. Food and Drug Administration, 2018). This statement is to the point of matters. The larger the treatment effect, the more opportunity and potentially even the more need for a less strict design. While an RCT may be appropriate for moderate treatment effect differences, it may also be too much of a good thing when the anticipated treatment effect size is large. In this case, research designs using external data are likely to appropriately contextualize findings from a SAT regardless of pre-specification. However, there is a valid question whether an ECT is likely to have this information about an anticipated large treatment effect available. Because the trial is yet at the planning stage, other data hinting at a large treatment effect may not always be existing. If indeed no information of an anticipated large treatment effect is available, no ECT design should be set-up as per FDA. This could mean in practice that ECT design opportunities are limited, while the ECC study design may constitute a more frequent use case.

Of note, terminology considerations have also relationships to established frameworks like the estimand framework (ICH E9(R1) Expert Working Group, 2021) and the target trial emulation framework (Hernán et al., 2022), which also aim to enhance clarity of planned or conducted research, aligning study design and analysis according to the underlying scientific question and to clarify the meaning of treatment effects, causal contrasts and applied methods.

As a conclusion, we have proposed a framework of nomenclature, which is applicable for research projects involving external data. Only a precise and consistent use of terminology will most effectively safeguard from unintended implications, inaccurate perceptions, and misguided mindsets.
